# A Full-Scale Experimental Investigation of Utility Poles Made of Glass Fibre Reinforced Polymer

**DOI:** 10.3390/ma14237398

**Published:** 2021-12-02

**Authors:** Mirosław Broniewicz, Filip Broniewicz, Elżbieta Broniewicz

**Affiliations:** Faculty of Civil Engineering and Environmental Sciences, Bialystok University of Technology, 15-351 Bialystok, Poland; f.broniewicz@doktoranci.pb.edu.pl (F.B.); e.broniewicz@pb.edu.pl (E.B.)

**Keywords:** sustainable construction, GFRP lighting poles, flexural behaviour, composite structures

## Abstract

Utility poles made of glass fibre-reinforced polymer (GFRP) are becoming increasingly common in European countries. Therefore, it is necessary to accurately examine their structural properties to ensure the integrity and safety of the poles. The purpose of this article is to compare the bending resistance of GFRP composite lighting columns obtained using European standard procedures with full-scale experimental tests. Several composite lighting columns were tested as part of the research study, and coupon tests were performed to assess the material properties required to calculate their bending resistance according to European Standard (EN) 40-3-3. The results obtained differed significantly. Furthermore, it was observed that the current standard rules for obtaining the resistance of GFRP poles based on the limit state method show a higher load capacity of the column in comparison to the capacity obtained from the tests.

## 1. Introduction

Utility poles can be made of wood, steel, concrete or fibre-reinforced composite materials. Fibre-reinforced composite poles made of composite materials represent a modern engineering solution in which sustainability and ecology play a significant role. These poles are made up of glass or carbon fibres arranged in various patterns and encased in polyurethane resin. The resins generally consist of vinyl ester, polyester and other epoxy compounds. The most frequent manufacturing methods of composite poles are pultrusion, filament winding and vacuum infusion. Composite poles are rapidly gaining acceptance throughout the utility industry, mainly due to the main advantages of glass fibre-reinforced polymer (GFRP) poles, such as exceptional strength-to-weight ratios, resistance to corrosion chemical attack, non-conductivity and long lifespans.

Composite poles represent a new group of poles that are becoming increasingly important in the lighting market. Concrete and metal poles still make up the vast majority of investments, but they are quite susceptible to adverse impacts of environmental conditions. On the other hand, composite elements are characterised by more significant durability. For this reason, as well as potential economic benefits, significant interest from investors in composite poles has arisen.

The composite pole is a modern and unconventional structural element, and for this reason, the method of calculating its capacity is not fully established. The anisotropy of the material, caused by the GFRP composite manufacturing method, results in difficulties in determining the stresses. The various strength characteristics do not allow the isotropic strength criteria to be applied. Moreover, the problem of the assessment of the failure models exists, e.g., buckling or loss of local stability, which depend on the shape and dimensions of the structure.

## 2. Experimental and Analytical Studies on GFRP Poles

Some problems make it difficult to understand the behaviour of GFRP poles fully. The most characteristic properties of GFRP elements, compared to conventional materials, are their high specific strength and low stiffness. Due to this, many elements fail because of buckling phenomena, either local or global. Global buckling of FRP profiles has been investigated by Barbero and Raftoyiannis [1] and Mottram [2], concluding that local buckling should also be considered in the case of thin-walled beams. There is much research on the topic of local buckling of fibre reinforced polymer (FRP) beams, including papers by Correia et al. [3,4], Bank [5] and Pecce [6], who researched GFRP profiles.

Vito et al. [7] published a paper on composite smart poles, analysing their strength and failure modes with the Finite Element Method. One of the conclusions was that a safe and functional GFRP shaft is 37–80% lighter than its steel counterpart. A similar study [8] was conducted for GFRP utility poles, finding optimum cross-section dimensions to satisfy the ASTM strength criteria. Madenci et al. [9] have investigated the topic of pultruded FRP profiles buckling and their free vibration. There seems to be an agreement that GFRP elements behave differently under loading than elements made from isotropic materials. 

Kollar [10] stated that axial compressive stresses might cause local buckling in closed cross-section GFRP elements. One of the features of lighting poles is an inspection hole placed near the bottom of the pole. It has been proven that notched FRP laminates tend to fail under compression due to the micro buckling of fibres in the vicinity of the notch [11,12]. Moreover, the compressive strength of the glass–epoxy composite is usually lower than its tensile strength [13]. Therefore, in the case of bending with the pole’s inspection hole side being its compressed side, there is a possibility of failure due to local buckling in the vicinity of the inspection hole.

There is little research on GFRP tapered poles with circular cross-sections in comparison to FRP beams research. A team of researchers from the University of Sherbrooke [14] carried out a project investigating the full-scale flexural behaviour of fibre-reinforced polymer tapered poles manufactured by filament winding technique. They discovered that local buckling in the vicinity of the inspection hole is the dominant failure model of these poles. The team presented new design criteria for composite poles that include both the geometric and material properties of the pole [15].

Saboori and Khalili [16] analysed tapered FRP transmission poles using the Finite Element Method. They used a second-order shell element and first-order shear deformation theory, comparing their results to those obtained from the analytical method. The study results suggest that when utilising more rigid fibres at the inner and outer laminas of the pole cross-section, a quasi-sandwich structure is formed that increases the performance of the FRP poles. They also performed a transient dynamic analysis of a GFRP pole [17]. Urgessa and Mohamadi [18] conducted further studies on such poles using ABAQUS software. This study concluded that the maximum stress in the FRP composite column increases with fibre orientation up to 45° from the axial direction and then decreases when increasing the fibre orientation to 60°.

The aim of recently conducted studies on GFRP tapered poles [19] was to give guidelines on calculating the effective flexural modulus of the poles. It was shown that the successful calculation of the effective flexural modulus requires detailed knowledge of the material, especially its fibre-to-matrix ratio and the material properties of the matrix and the fibres. 

The European standards relevant to GFRP poles design are EN 40-7 [20] and EN 40-3-x [21,22,23]. The EN 40-7 [20] standard mentions the possibility of failure due to buckling (Annex B); however, there are no specific calculation models. Therefore, the burden of providing the pole’s buckling strength is placed on manufacturers.

The literature review indicates that the problem of GFRP poles failing due to local buckling in the vicinity of the inspection hole is severe and covers a wide range of column cross-sections. It is not limited to certain specific column dimensions and affects both slender and thick poles. However, there is still too little research on this phenomenon to fully understand the pole’s behaviour and present designer-friendly procedures to assess its bending strength.

This paper aims to examine the behaviour of GFRP lighting poles under lateral loading to discover the primary model of failure and its ultimate bending strength. Firstly, coupon tests were conducted to examine the material’s mechanical properties, including bending and tensile testing of samples. These properties are necessary to evaluate the accuracy of the computational models presented in both the literature and the standards. Next, full-scale tests of the poles were carried out to obtain their failure mechanisms and ultimate bending capacity. The material properties obtained from the coupon tests were used to calculate the ultimate bending strength according to the EN 40-7 standard. Finally, both ultimate bending strengths (experimental and calculational) were compared. The comparison allows for an assessment of the accuracy of current design procedures. In addition, the authors’ comments on the shortcomings of the current standard and possibilities to improve it are presented. These comments are a prelude to designing a new procedure for calculating the load capacity based on test results.

## 3. Material Properties Tests

In order to proceed with the calculational verification of the poles, it was necessary to obtain several mechanical properties of the material. To calculate the pole’s ultimate bending strength, the EN 40-7 standard requires five independent properties: *f_y_*—characteristic yield strength of the material, *E_1_*, *E_2_*—the characteristic modulus of elasticity of the material in, respectively, the longitudinal and transverse direction, and the corresponding Poisson’s ratios *v_12_* and *v_21_*. These properties were obtained from tensile and flexural tests.

### 3.1. Tensile Test

The method and conditions of the tensile tests are described in ISO 527-4 [24]. The conducted tests aimed to determine the tensile properties of orthotropic fibre-reinforced material, such as the tensile strength *σ_m_*, tensile failure strain ε*_m_* and modulus of elasticity *E*.

**Figure 1:**
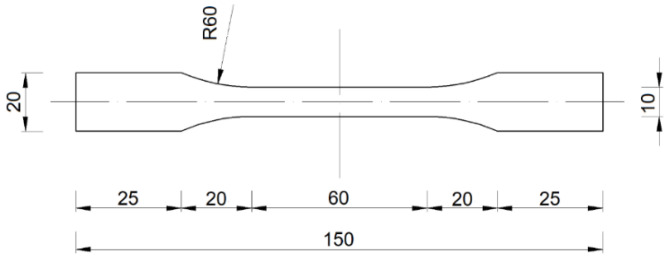
Tensile test specimen dimensions in mm (in accordance with [24]).

According to ISO 527-1 [25], the minimum number of test specimens was five. The values of the mechanical properties were determined with a 95% confidence interval. According to ISO 527-4 [24], the type of specimen used was 1B (Figure 1).

**Figure 2:**
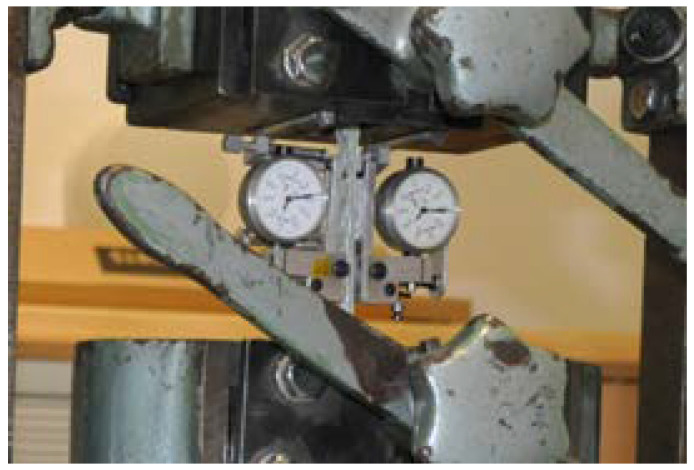
Specimen No. 4 during the tensile test.

The specimens were cut out from the columns’ shafts to make the longitudinal axis of the specimen runs parallel to the axis of the pole. During the tests, the specimens were placed in the testing machine, ensuring that the specimen’s longitudinal axis closely coincides with the longitudinal axis of the testing machine. The tensile test was performed on a Heckert ZD 10/90 static testing machine (Figure 2) manufactured by the company WMW AG, Erfurt, Germany. The tests were carried out in the range of up to 100 kN, with a minimum increment value of 200 N; the test temperature was +19 °C.

**Figure 3:**
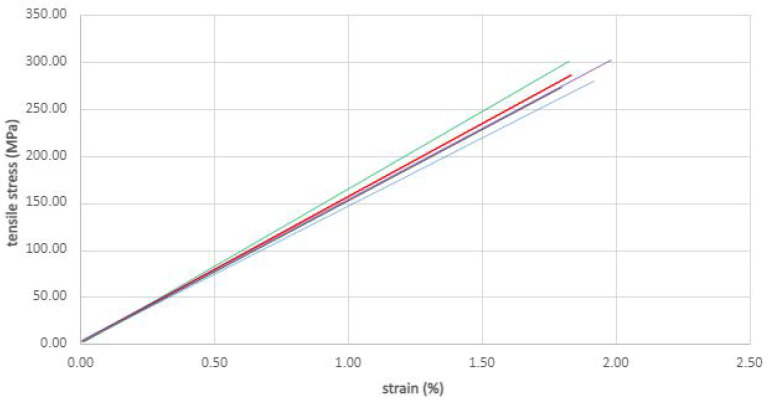
Coupon tensile tests: strength–strain curves.

During the tests, the specimen was loaded with tensile force along the main axis at a constant speed of 0.18 mm/s until failure. The values of the tensile force and the elongation of the measurement base of the tested specimen were recorded automatically, which allowed the stress–deformation curve characterising the tested material to be obtained (Figure 3).

A total of seven specimens were examined, five of which were accepted for further statistical processing. Two tested samples were rejected as their results were considered unreliable due to the elongation measuring device slippage on the sample surface. The values of the tested material characteristics were determined with the confidence level of 95%, required by ISO 2602 [26].

**Figure 4:**
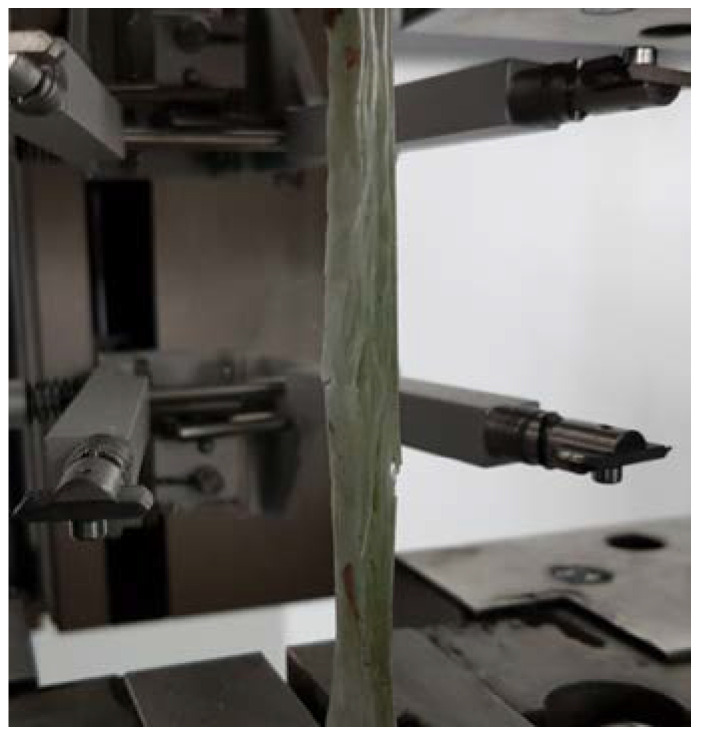
Ruptured specimen after static tensile test.

The estimated Young’s modulus of elasticity in tension was obtained with linear regression of the experimental strength–strain curves corresponding to extensions ranging from 0.05 to 0.25. All specimens exhibited tensile failure in narrow gauge length (Figure 4).

**Figure 5:**
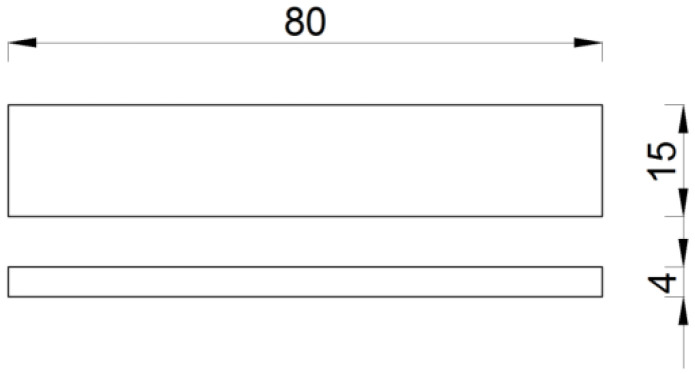
Flexural test specimen dimensions in mm (in accordance with [27]).

The brittle fracture was the failure mechanism of all tested GFRP samples. The tested GFRP specimens failed suddenly at the ultimate stress in the tensile test, and they all failed in the same manner, that is, fibre rupture. Due to surface debonding of the fibres and matrix, the failure occurred with abrupt longitudinal delamination of the laminae at the centre. As a result, as seen in Figure 5, broken fibres were divergent and fan shaped.

The values obtained for *E_1_* and *E_2_* were similar, concluding that the material can be considered transversely isotropic. Transversely isotropic materials are standard practice in composite manufacturing elements that do not need an extensive design process and differentiation in material properties depending on the direction. Therefore, it was assumed in the further calculation that *E_1_* = *E_2_* and only *E_1_* is presented in Table 1.

The results of the static tensile test average values and standard deviation values are shown in Table 1. 

### 3.2. Flexural Test

The flexural behaviour of the GFRP material was tested by applying bending force at a constant speed to the standardised specimens until fracture was reached. This method was used to test the flexural strength *σ_fM_* and flexural stress at break (rupture) *σ_fB_*. The bending test was conducted following the requirements of PN-EN ISO 14125 [27]. The dimensions of the specimens met the requirements of ISO 14125 [27] for material class II (Figure 5).

**Figure 6:**
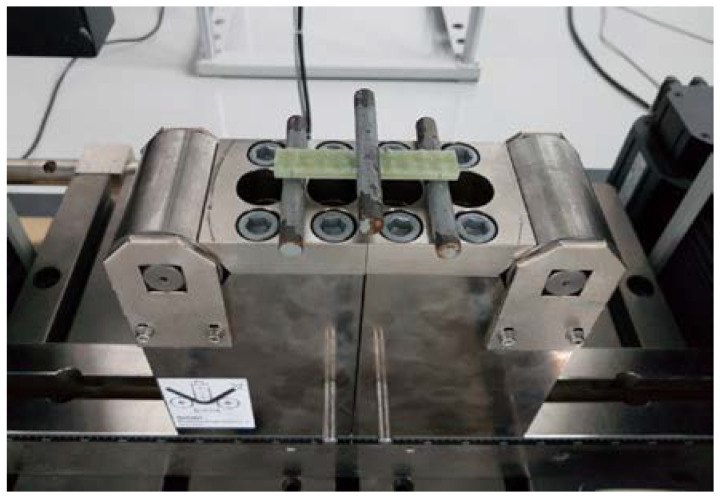
Specimen No. 2 during the three-point flexural test.

The test consisted of subjecting the specimens cut from the GFRP column material to three-point bending until tensile failure of the bottom fibres. The distance between the support members was *L* = 64 mm, and the loading speed was 2 mm/min. The rounding radii of the supports and central loading members were *R_1_* = *R_2_* = 5.1 mm. The flexural test was carried out on a ZD 10/90 static testing machine (Figure 6). Mid-span displacements were measured with dial indicators with a 0–10 mm measurement range and a resolution of 0.01 mm.

All the specimens behaved elastically during loading, up to failure. This material feature is present because undamaged glass fibres retain their linear elastic behaviour during deformation.

The characteristic values of the flexural strength *σ_fM_* and flexural stress at break *σ_fB_* are presented in Table 2.

## 4. Cantilever Beam Static Bending Test

### 4.1. Manufacturing Method

The filament winding technology was used to produce the lighting poles in this investigation. In the technological process of manufacturing elements, several fibre rovings are drawn from a sequence of creels and deposited in a bath containing liquid resin, catalyst and additional chemicals such as colours and UV retardants. The guides or scissor bars were positioned between each creel and a resin bath controlled fibre tension. The resin-impregnated rovings were drawn through a wiping device at the end of the resin tank, removing excess resin from the rovings and adjusting the resin coating thickness surrounding each roving.

The wiping device consisted of a series of squeezing rollers, with the top roller’s location being utilised to manage the resin content and the tension in fibreglass rovings. The final procedure allows for improved resin content control. The filament wound mandrel was exposed to curing and post-curing procedures after winding, during which the mandrel is constantly rotated to preserve resin content homogeneity around the circle. After curing, the result was mechanically extracted from the mandrel.

The filaments used are glass fibres with a filament diameter of 20 μm impregnated in an unsaturated polyester (UP) resin bath. The helical winding method was used, with a winding angle of 60° relative to the tube’s longitudinal axis.

### 4.2. Test Setup

The objective of cantilever beam testing was to measure the failure phenomenon of the pole. Specifically, the location, mode of failure and ultimate bending strength were to be determined. The poles subjected to testing were 3, 5 and 7 m long, with their other dimensions depending on the length of the pole (Table 3).

The laminate thickness was measured in the vicinity of the inspection hole. The measurements varied. Therefore, five measurements were taken for each column, and both the standard deviation and confidence intervals were calculated [28]. The results are shown in Table 4.

The research covered nine poles. All the prototypes investigated in this study are single segment poles with no extra reinforcement provided around the hole located under the ground line. They were made of an E-type glass fibre composite and epoxy resin. All the holes were cut at the manufacturer’s site after the poles were fabricated. For the 3 m and 5 m poles, the dimensions of the inspection hole were 75 mm × 200 mm, and the hole was located 700 mm above the ground line. For the 7 m poles, they were 85 mm × 300 mm and 600 mm, respectively. The dimensions of the cross-section of the columns depended on the height of the post. 

**Figure 7:**
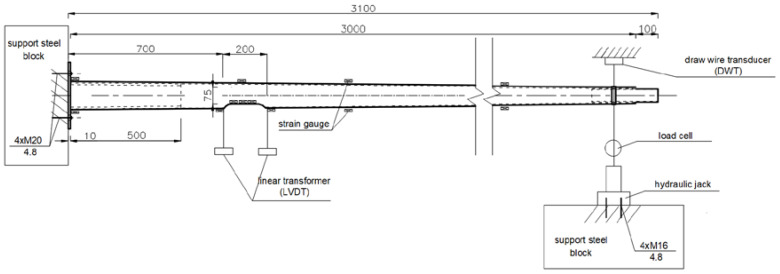
Schematic drawing of the full-scale test setup for 3.0 m poles (dimensions are given in mm).

A test setup was prepared according to the recommendations of EN 40-3-2 [22]. A schematic drawing of the full-scale test setup for 3 m poles is presented in Figure 7.

**Figure 8:**
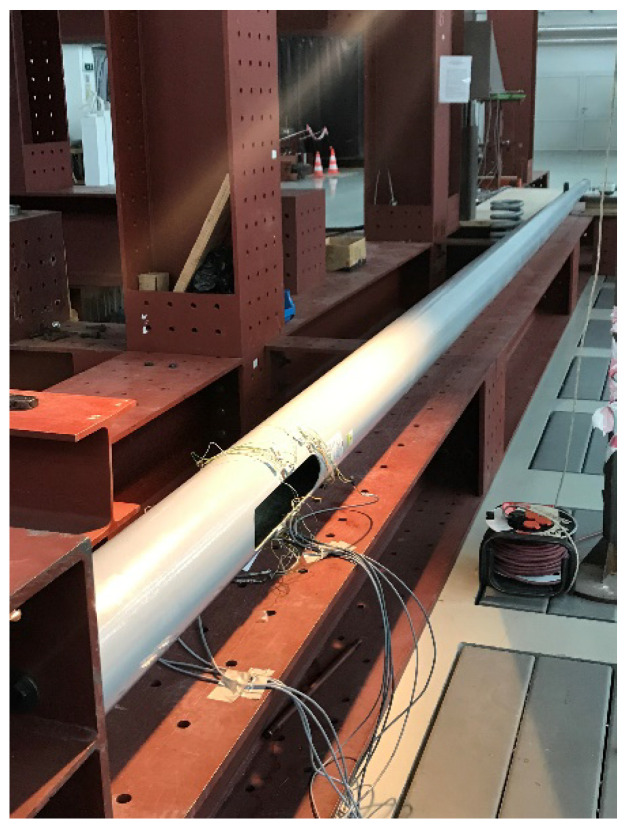
Pole 7b placed on the test setup.

The test setup involved a support block, approximately 600 mm wide and 800 mm high, which was used to rigidly fix the column with an anchor bolt (Figure 8). The poles were attached to the test stand with four M20 screws and supported in 2/3 of their length on a sliding support to eliminate the effect of the column’s weight. The support was based on sliding bearings, which eliminated friction between the surface of the column and the support. The single tension load was applied in a slow quasi-static manner using a hydraulic jack mounted on a steel block, placed 0.5 m from the top of the poles.

The pole was loaded in three stages, in accordance with [22]. In the second stage, the ultimate limit state test was carried out. The column was loaded up to the design value of the design load, the horizontal deflection was recorded and critical cross-sections were checked for any signs of damage. 

The last stage was the ultimate failure test. First, the column was further loaded until it was destroyed. Then, permanent deformation of the end of the column and the force at failure were measured. The observed failure mode was local buckling of unstiffened walls in the vicinity of the inspection hole in the pole’s compression zone.

### 4.3. Instrumentation

Electric strain gauges were used to measure the longitudinal deformation of the column. They were located on the compression and tension side of the column, at the base, around the opening and every 1/4 of the column length. All specimens were instrumented symmetrically towards the midpoint by seven strain gauges TFs 10/120 with ohmic resistance 120 ± 0.2% W, produced by TENMEX. The strain gauges were mounted on the compression side of the poles at eight different locations. Three strain gauges were mounted at each location to measure the strains in the longitudinal, circular and 45 degrees off-axis directions.

The displacement was measured at the critical cross-sections of the pole, which are:

- the lower edge of the inspection hole;
- the upper edge of the inspection hole;
- the lantern fixing point.

The displacement of the column at the edge of the opening was measured using linear variable differential transformers (LVDTs). The deflection of the top parts of the FRP poles was measured with a draw wire transducer (DWT) located under the load application point. The load was monitored through an electronic standard stainless steel high-capacity load cell. To automatically read, store and transmit the load, LVDTs, DWTs and strain gauge measurements, an automatic data acquisition system (ADAS) was used.

### 4.4. Failure Modes

**Figure 9:**
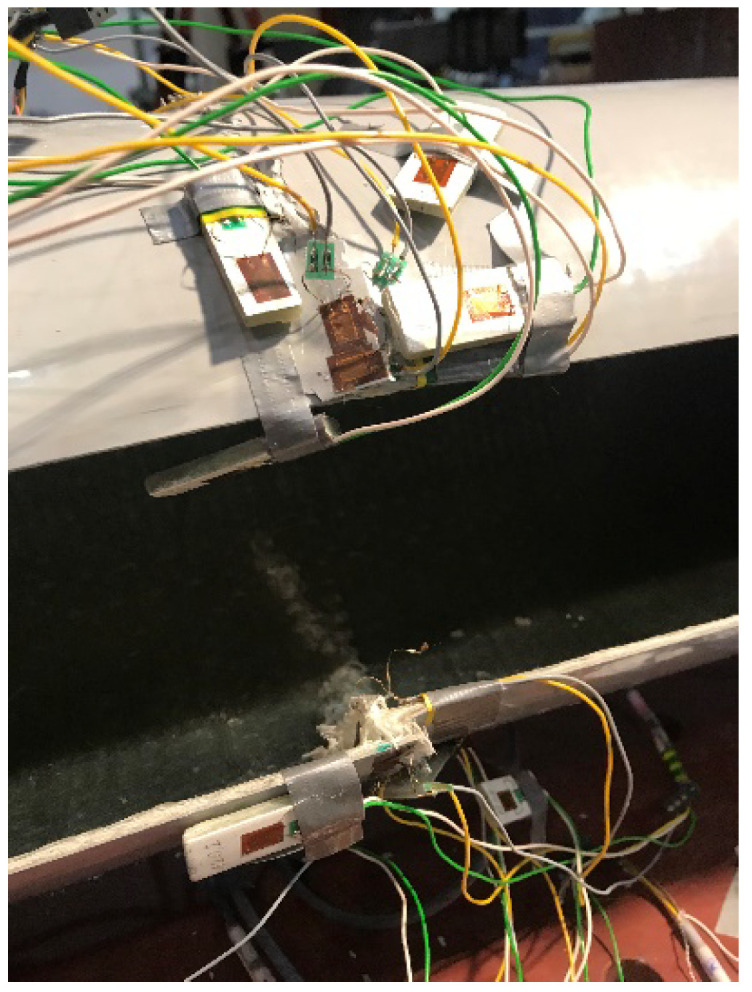
Total failure view of the pole in the area of the inspection hole.

In the test specimens, the inspection hole was located on the compression side of the column. All the tested elements were damaged by local buckling and finally cracking or crushing the resin or fibres in the middle area of the inspection hole (Figure 9). This failure happened suddenly after the local buckling of the walls in the vicinity of the inspection hole’s edge. During the tests, no other signs of damage to the tested elements were found.

The material failure was preceded by the ovalisation of the shaft and buckling of the longer edges of the inspection hole. The buckling of the sides was caused by high normal compressive stresses parallel to the column axis. The buckling resulted in the exceedance of the compressive stress limit of the pole’s inner lamina. This step can be described as a first-ply failure. The increase of load with the simultaneous reduction of load-bearing laminae resulted in the failure of the whole laminate on one side of the hole. This failure caused a redistribution of stresses, which resulted in a similar failure on the other side of the inspection hole.

**Figure 10:**
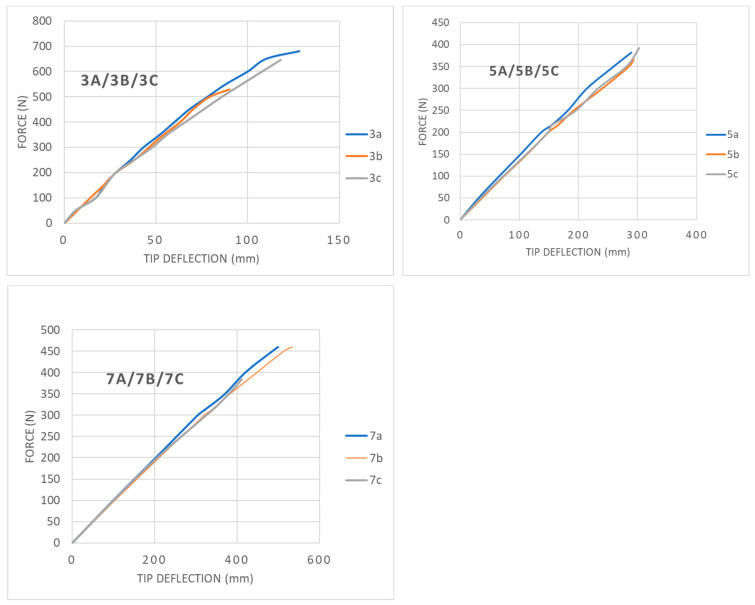
Force-tip deflection curves for tested poles.

The maximum tip deflections of the poles at the time of material failure are presented in Table 5. Figure 10 show the load–deflection curve for the tested poles up to the moment of failure. The load–deflection curve for the pole’s tip was linear in the initial stage. In the near-failure state, one could observe a slight decrease of tangent stiffness caused by gradual fibre breakage. The moment of column failure was characterised by an increase in column deflection with no increase of the applied force and a visible rupture of the composite fibres.

## 5. Analytical Calculation of Critical Bending Moment

**Figure 11:**
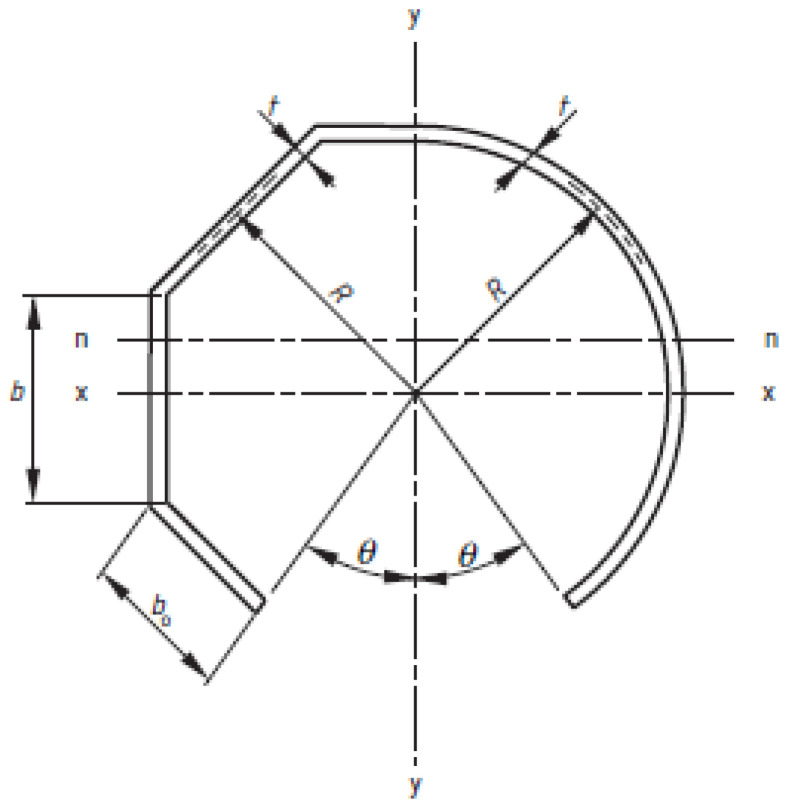
Cross-section of the pole at the inspection hole level.

The calculation of the critical bending moment value was performed using the procedure taken from [23]. This procedure is relevant in the European Union when obtaining the CE certificate required to put the pole on the market. It is based on the limit state method, in which the values of bending moments in critical sections of the pole caused by design loads must be lower than the bending strength of a given section. The bending strength of a given cross-section depends on its geometric and material properties and is calculated using the following equation:

(1) $$M_{ux}=\frac{f_{y}\cdot g\cdot \phi_{3}\cdot Z_{pn}}{\gamma_{m}},$$

where $\;Z_{pn}$ is the plastic modulus of inspection hole cross-section, $\phi_{3}$ is the factor depending on the dimensions of a pole and its mechanical properties, g is the shape factor, $f_{y}$ is the pole material’s tensile strength and $\gamma_{m}$ is a partial material factor. The plastic modulus is calculated with the following equation:

(2) $$Z_{pn}=2\cdot F\cdot \;R^{2}\cdot t\cdot \cos\left(\frac{\theta }{2}\right)\cdot (1-\sin\left(\frac{\theta }{2}\right),$$

where F is the shape factor, R is the circular cross-section radius, t is the wall thickness, and θ is the half-angle of the door opening. Parameters used in the calculation of bending strength are presented in Table 6. The mechanical properties were obtained from coupon tests. Their values were used to apply the calculation procedure. Since all the tested columns failed near the centre of the inspection hole, the procedure was performed to calculate the bending strength (ultimate bending moment) for the cross-section at the inspection hole’s centre level. Moreover, bending surrounding the axis (*x-x* axis in Figure 11) perpendicular to the axis passing through the hole’s centre was assumed. Unfortunately, the standard PN EN 40-3-3 [23] does not consider the direction of the pole bending (i.e., whether the hole is on the compression or tension side of the cross-section).

## 6. Comparison between Experimental and Theoretical Results

The poles did not reach the bending strength value calculated using the [23] standard procedure during the test. Table 7 show a comparison of the values of the experimental ultimate bending moments *M_B_* with their design bending resistances *M_ux_* calculated by the relevant standard at the inspection hole level.

Considering the uncertainty of laminate thickness measurements (Table 4), the bending strength (*M_ux_*) was calculated for the lower and upper expected thickness values.

The comparison shows that the bending resistance value of the columns in the middle of the inspection hole obtained from experimental tests is between 0.26 and 0.39 of the bending resistance values calculated in accordance with the standard [23] and is therefore up to three times lower in many cases. The standard does not consider the pole’s failure by local buckling at the inspection hole. The possibility of this form of column damage is mentioned in the standard [23], but without providing specific recommendations for calculating the pole’s bending strength. Therefore, it seems that the European Standard EN 40-3-3 does not successfully calculate the real strength of the pole with an inspection hole. 

## 7. Conclusions

This article presents a study on the behaviour of composite columns under bending load. Material samples were subjected to flexural and tensile tests to obtain the mechanical properties of the pole’s material. The tests indicated linear ductility up to the breaking phase. Moreover, they were characterised by low stiffness typical of GFRP elements.

The bending resistance of the columns and their damage modes were investigated. The research showed that the deformation of the column increased linearly until it was close to failure (near-failure zone). All the tested poles failed in the same way, i.e., by buckling of inspection hole’s long free edges. This is due to the low stiffness modulus of the column material and the reduction of the cross-section by the inspection hole. The 3 m long columns’ ultimate force was ~600 N; for 5 and 7 m long columns, the force was~400 N. This failure mode of the poles is not predicted by the EN 40-3-3 standard, which contains the procedure for calculating the strength of the lighting composite columns.

The calculation procedure included in the EN 40-3-3 standard was carried out to check the value of the expected ultimate bending moment. For this purpose, the dimensions of the columns were obtained from the direct measurements. Then, the mechanical properties of the GFRP material were determined through a series of tests on coupons cut from undamaged parts of the columns. All these values were used in the calculation procedure. The values of the ultimate bending moment (UBM) for the columns obtained using the procedure were always significantly higher than the value of the UBM obtained from the tests. The design values of the UBM obtained according to the standard procedure were two and three times higher than the values obtained experimentally. The design procedure analysis reveals that the influence of the inspection hole is only considered in the design standard as a local reduction of the cross-section that reduces the cross-plastic section’s modulus. The possibility of compressed inspection hole walls’ buckling is not taken into account. This fact indicates that the procedure is not adjusted to the actual behaviour of the columns. 

## Figures and Tables

**Table 1:** Results of tensile tests (averages and standard deviations).

Mechanical Properties	Average Value	Standard Deviation	5% Quantile
Tensile strength σ_m_ (MPa)	281.5	10.3	279.5
Strain at strength ε_m_ (%)	1.87	0.078	1.82
Modulus *E_t_* (MPa)	22,490	1631.8	21,320

**Table 2:** Results of flexural tests (averages and standard deviations).

Mechanical Properties	Average Value	Standard Deviation	5% Quantile
Flexural strength *σ_fM_* (MPa)	254.1	18.9	230.3
Flexural stress at break *σ_fB_* (MPa)	243.3	19.6	217.9

**Table 3:** Dimensions of tested poles.

Pole Identification	Length L (mm)	Bottom/Top Diameters (mm)	Mean Bottom Thickness (mm)	Inspection Hole		
				Dimensions (mm)	Location A (mm)	Positioning
3a	3015	128/46	4.8	205 × 76	705	Compression
3b	3014	128/47	4.9	205 × 75	705	Compression
3c	3014	129/47	4.3	205 × 76	704	Compression
5a	5005	129/47	5.2	205 × 76	704	Compression
5b	5003	129/46	5.6	204 × 76	705	Compression
5c	5005	128/47	5.5	205 × 76	705	Compression
7a	7008	149/73	5.9	299 × 86	608	Compression
7b	7012	150/73	6.1	300 × 85	607	Compression
7c	7008	150/72	5.9	300 × 85	603	Compression

**Table 4:** The measurements of the thickness of poles (mm).

Pole Identification	Measurements					Parameters		
	1	2	3	4	5	Average Value (mm)	Standard Deviation	Confidence.T
3a	4.6	5.0	4.9	4.8	4.8	4.8	0.15	4.64–5.00
3b	4.5	4.9	4.7	5.1	5.2	4.9	0.26	4.61–5.15
3c	4.2	4.2	4.6	4.1	4.5	4.3	0.22	4.05–4.59
5a	5.3	5.5	5.6	4.8	5.0	5.2	0.30	4.95–5.53
5b	5.2	5.6	5.5	5.7	5.8	5.6	0.23	5.27–5.85
5c	5.5	5.2	5.1	5.6	5.9	5.5	0.32	5.06–5.86
7a	5.8	6.0	5.6	5.9	6.3	5.9	0.23	5.68–6.16
7b	6.2	6.1	5.8	5.9	6.4	6.1	0.24	5.78–6.38
7c	5.4	6.2	5.9	5.9	6.1	5.9	0.31	5.52–6.28

**Table 5:** The experimental ultimate failure load and top deflection.

Pole Identification	Length L (mm)	Force F_B_ (N)	Bending Moment at Failure M_B_ (Nm)	Tip Deflection at Failure u (mm)
3a	3015	532	909	128
3b	3014	683	1167	90
3c	3014	645	1102	118
5a	5005	382	1412	289
5b	5003	366	1353	293
5c	5005	393	1454	303
7a	7008	463	2649	499
7b	7012	459	2651	533
7c	7008	385	2216	412

**Table 6:** Parameters used in the calculation of bending strength.

Pole Identification	Length L (mm)	Mean Radius of the Cross-Section R (mm)	Plastic Modulus of the Cross-Section about the n-n Axis (mm^3^)	Bending Strength at the Inspection Hole Level M_ux_ (Nm)
3a	3015	50.6	26,202	3283 ± 123
3b	3014	50.7	26,833	3401 ± 188
3c	3014	51.4	24,608	2861 ± 179
5a	5005	55.3	36,875	4716 ± 261
5b	5003	55.0	39,299	5240 ± 273
5c	5005	54.7	38,192	5047 ± 370
7a	7008	67.4	67,410	7792 ± 316
7b	7012	67.8	70,423	8303 ± 410
7c	7008	67.9	68,246	7862 ± 506

**Table 7:** Comparison of experimental and calculational pole’s bending strengths.

Pole Identification	Bending Moment at Failure *M_B_* (Nm)	Bending Strength *M_ux_* (Nm)	*M_B_*/*M_ux_*
3a	909	3283 ± 123	28% ± 1%
3b	1167	3401 ± 188	34% ± 2%
3c	1102	2861 ± 179	39% ± 2%
5a	1412	4716 ± 261	30% ± 2%
5b	1353	5240 ± 273	26% ± 1%
5c	1454	5047 ± 370	29% ± 2%
7a	2649	7792 ± 316	34% ± 1%
7b	2651	8303 ± 410	32% ± 2%
7c	2216	7862 ± 506	28% ± 2%

## Data Availability

Data Sharing is not applicable for this article.
